# A Case Report of Erythema Multiforme Secondary to Atorvastatin Use

**DOI:** 10.7759/cureus.52175

**Published:** 2024-01-12

**Authors:** Ann M Chacko, Giselle Carrero, Shweta Akhouri

**Affiliations:** 1 College of Medicine, Florida International University, Herbert Wertheim College of Medicine, Miami, USA; 2 Clinical Pharmacy, Nicklaus Children's Health System, Miami, USA; 3 Family Medicine, Florida International University, Herbert Wertheim College of Medicine, Miami, USA

**Keywords:** statin induced, medication side effects, targetoid lesion, erythema multiforme minor, cutaneous rash, statin safety, statin side effects, erythema multiforme (em)

## Abstract

Erythema multiforme (EM) is a rare and typically self-limited mucocutaneous reaction known to present secondary to various triggers, with the most common being from an infectious etiology. Medications account for a small percentage of EM cases. Here, we report a case of a 55-year-old female who presented to her primary care physician with a circular rash on the palm of her right hand, which she noticed five days after being started on atorvastatin due to right branch retinal artery occlusion. The rash was identified as case of non-photoinduced EM associated with atorvastatin use presenting solely on the palmar aspect of the patient's hand and resolving four days after discontinuation of the medication. Current literature only describes photoinduced cases of EM secondary to statin use making this case unique, and it provides important insights about considering alternative lipid-lowering treatment options for patients with recurrent or persistent cases.

## Introduction

Erythema multiforme (EM) is a rare, acute, immune-mediated, and typically self-limited mucocutaneous reaction that can be isolated, recurrent, or persistent [1,2]. Most commonly, it presents symmetrically on the acral surfaces as targetoid lesions with concentric color variation that may or may not be associated with mucosal involvement of the genital, oral, or ocular surfaces [1,2]. EM major is the term used for EM with mucosal involvement (and possible systemic symptoms), whereas EM minor does not involve the mucosal surface [2]. Although EM is often self-limiting, if left untreated, it may result in complications, such as pruritis or burning of the skin, pain caused by mucosal erosions, swelling of the hands and feet, post-inflammatory hyperpigmentation, and, rarely, esophageal stricture formation [2]. In addition, those with ocular mucosal involvement may develop uveitis, conjunctival scarring, keratitis, or even blindness [2]. A myriad of precipitating factors for EM have been documented in the literature, such as infections, medication use, autoimmune disease, immunization, radiation, and malignancy, but only a few are well described [1-4]. We report a case of a 55-year-old female who presented to the primary care clinic with a targetoid, tender rash on her right palm after recently starting atorvastatin. The patient was diagnosed with EM minor, which successfully resolved after discontinuing the medication. As we continue to learn more about the various precipitants of EM, primary care providers play an essential role in recognizing the condition and identifying the various causes associated with it to minimize future recurrences and mitigate complications. It is integral to contribute to the current literature on uncommon precipitants of EM, such as atorvastatin, which is a commonly prescribed medication.

The etiology of EM is thought to be due to a cell-mediated immune reaction, and 90% of cases have been attributed to infections, with the most common cause being herpes simplex virus (HSV), followed by mycoplasma pneumoniae [1-3]. Medications, including antiepileptics, non-steroidal anti-inflammatory drugs, and antibiotics, account for less than 10% of EM cases, while barbiturates and statins contribute to a smaller percentage [1-2,4]. Interestingly, HSV-associated EM is thought to be mechanistically distinct from drug-induced EM, as lesions associated with HSV have tested positive for interferon gamma, whereas lesions associated with drugs are associated with the expression of tumor necrosis factor-alpha [1,5]. This may be an important consideration when considering treatment options that can target specific cytokines.

Although there is growing literature on the different causes of EM, there are only a few reported cases of statin-induced EM [6,7]. There have been three reported cases of photoinduced EM affecting the face, forearms, and/or dorsum of the hands secondary to the use of simvastatin, pravastatin, and atorvastatin [6,7]. With statins being one of the first-line cholesterol-lowering agents, it is important to recognize EM as a possible side effect. We present a case of a non-photoinduced EM associated with atorvastatin use that presented solely on the palmar aspect of a patient’s hand, a location that has not yet been reported on in the literature.

## Case presentation

A 55-year-old female with a past medical history of hypertension, obesity, and tobacco use presented to the primary care clinic due to a “rash” on her right palm that she noticed that same morning. The rash was described as dark, circular, and tender to palpation. The patient referred to the package insert for atorvastatin and noted it was listed as a side effect.

Six days prior to this episode, the patient was sent to the emergency department by her ophthalmologist due to concerns of right-sided retinal artery branch occlusion after the patient reported vision loss in her right upper nasal visual field. Magnetic resonance imaging (MRI) brain showed mild, chronic small vessel ischemic changes. Magnetic resonance angiography (MRA) of the neck and brain, echocardiogram with bubble study, and electrocardiogram (EKG) were all normal. Erythrocyte sedimentation rate (ESR) and C-reactive protein (CRP) were normal; however, her lipid panel showed a low density lipoprotein (LDL) of 190 mg/dL (normal: less than 100mg/dL). The patient was discharged the next day on aspirin 81 mg by mouth once daily and atorvastatin 80 mg by mouth once daily with a diagnosis of right branch retinal artery occlusion.

**Figure 1:**
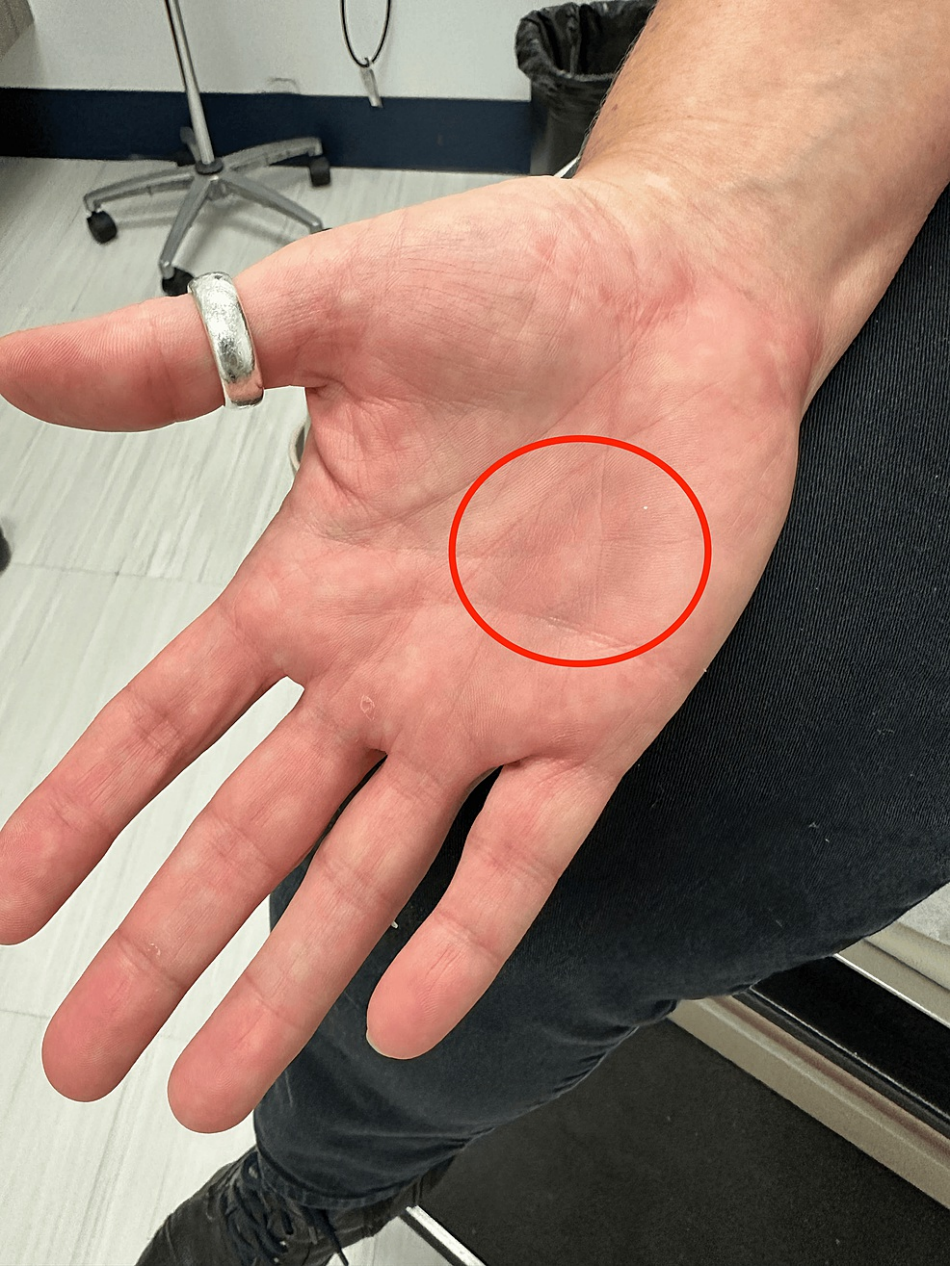
Erythema multiforme on the right palm of patient five days after initiation of atorvastatin

Upon presentation in the primary care office five days after discharge, the patient was afebrile (97.3 degrees F) and normotensive (120/95 mmHg). Her pulse (90/min), respiratory rate (12/min), and oxygen saturation (98% on room air) were all within normal limits. On physical exam, the patient was alert, well developed, well nourished, and in no acute distress. On skin examination, a 2x3 cm hyperpigmented targetoid lesion with central clearing was noted on the palmar aspect of the right hand, as seen in Figure 1. The lesion was mildly tender to palpation. No other rashes were noted elsewhere including the mucous membranes. The remainder of the physical examination was normal.

Her current medications included amlodipine 5 mg by mouth once daily, aspirin 81 mg by mouth once daily, and atorvastatin 80 mg by mouth once daily. She had a history of smoking ½ pack per day for 20 years and quit six years ago. She consumes alcohol socially. She denied any illicit drug use. Her family history is unknown as she is adopted.

A clinical diagnosis of EM minor was made, and atorvastatin was discontinued to assess for improvement. The patient confirmed that the rash disappeared within four days of discontinuing atorvastatin. The subsequent plan was to change her high-intensity statin to a moderate intensity, but the decision was deferred to her cardiologist. The patient has not returned for follow-up since.

## Discussion

Statins are widely prescribed medications used to lower cholesterol and reduce cardiovascular morbidity and mortality. The primary mechanism of action of statins involves inhibiting the rate-limiting enzyme of cholesterol biosynthesis - HMG-CoA (3-hydroxy-3-methylglutaryl coenzyme A) reductase [8]. Although statins are widely used and have substantial evidence for their benefit, many individuals still discontinue the therapy due to side effects. Observational studies show that statin intolerance occurs in 10-15% of patients, whereas clinic data show that it may be as high as 30% [8].

The most prevalent and well-documented adverse effects are statin-associated muscle symptoms (SAMS), which include myalgia, myopathy, myositis with elevated creatinine kinase, and rhabdomyolysis [8]. Other side effects that have no established validity but have been reported include neurocognitive and neurological effects, renal toxicity, liver toxicity, and effects that involve the urogenital, reproductive, and gastrointestinal systems [8]. Dermatological side effects of statins are not common, but conditions, such as cutaneous lupus erythematosus, bullous dermatosis, eczematous skin reaction, cheilitis, ultraviolet-B phototoxicity, photosensitivity, porphyria cutanea tarda, acute exanthematous pustulosis, and dermatomyositis-like syndrome, have been reported with statin use [9]. Based on our literature review, there are only a limited number of reports of statin-associated EM, and they have all been photoinduced [6,7].

In our case, although we cannot definitively prove the patient’s rash was caused by atorvastatin, resolution of the rash within four days of discontinuation suggests that this was the most likely etiology. Moreover, since the patient’s rash emerged on the palmar surface of her right hand, it is presumed to be non-photoinduced as the dorsal aspect of the hand is normally affected when photoinduced, making it a distinct case from the ones mentioned prior. In addition, the patient did not report any unusual or extended sun exposure at the time.

As there are limited reports of statin-induced EM, it is not possible to determine which statin is most likely to cause the reaction compared to other statins; however, this information would be helpful when deciding the next treatment options for individuals who develop such a side effect. In the case of a 49-year-old male who developed photoinduced EM, reintroduction of atorvastatin at a smaller dose resulted in the rash reappearing; hence, decreasing the dose itself may not be a promising solution [6]. More cases need to be reported in order to determine if there are certain statins that are more prone to causing EM so that alternative statins may be prescribed.

Although EM is typically benign and managed with anti-inflammatories for pain, if symptoms are recurrent or persistent, then alternative lipid-lowering therapies for those who develop EM secondary to statin use should be considered. Regardless of statin intolerance, lifestyle modifications with exercise, diet, and smoking cessation should be emphasized to reduce cholesterol levels and cardiovascular risk [10]. Since there is limited research on this topic, general recommendations for statin intolerance may be followed. The patient should be counseled on alternative lipid-lowering agents, such as ezetimibe, which has been shown to reduce LDL cholesterol levels by 20% [10]. If ezetimibe therapy is not sufficient to achieve LDL cholesterol goals, a fibrate or bile acid binding resin may be added to the regimen [10]. In addition, PCSK9 inhibitors have been approved in the United States and Europe for statin intolerance, with research showing that alirocumab and evolocumab result in LDL cholesterol reductions of more than 50% and have cardiovascular benefit [10]. Such alternative therapies should only be considered if a patient is truly intolerant to statins and develops EM after statin dose reductions and trying alternative statins. Until further evidence and research develop on this topic, it is up to the primary care physician to use a patient-centered approach when determining which next step to take.

## Conclusions

As additional research becomes available, our knowledge of the multiple factors behind EM grows. The emergence of statins as potential triggers for this immune response highlights the need for further investigations to identify which specific statins may be more likely to induce the reaction. In addition, exploring alternative cholesterol management strategies for individuals with atherosclerotic disease who cannot take statins due to EM is essential. It is integral that we report this case because our patient developed a non-photoinduced EM that resolved with the discontinuation of atorvastatin with no development of complications. Being aware that EM may be a possible side effect of statin use is crucial for primary care physicians in order to diagnose and treat patients in a timely and effective manner.
